# RNA N6-Methyladenosine Regulators Contribute to Tumor Immune Microenvironment and Have Clinical Prognostic Impact in Breast Cancer

**DOI:** 10.3389/fgene.2021.650499

**Published:** 2022-01-13

**Authors:** Lan-Xin Mu, You-Cheng Shao, Lei Wei, Fang-Fang Chen, Jing-Wei Zhang

**Affiliations:** ^1^ Hubei Key Laboratory of Tumor Biological Behaviors, Zhongnan Hospital of Wuhan University, Department of Breast and Thyroid Surgery, Hubei Cancer Clinical Study Center, Wuhan, China; ^2^ Hubei Provincial Key Laboratory of Developmentally Originated Disease, Wuhan University, Department of Pathology and Pathophysiology, School of Basic Medical Sciences, Wuhan, China

**Keywords:** m6a, tumor microenvironment, breast cancer, prognosis, risk model, molecular subtype

## Abstract

**Purpose:** This study aims to reveal the relationship between RNA N6-methyladenosine (m6A) regulators and tumor immune microenvironment (TME) in breast cancer, and to establish a risk model for predicting the occurrence and development of tumors.

**Patients and methods:** In the present study, we respectively downloaded the transcriptome dataset of breast cancer from Gene Expression Omnibus (GEO) database and The Cancer Genome Atlas (TCGA) database to analyze the mutation characteristics of m6A regulators and their expression profile in different clinicopathological groups. Then we used the weighted correlation network analysis (WGCNA), the least absolute shrinkage and selection operator (LASSO), and cox regression to construct a risk prediction model based on m6A-associated hub genes. In addition, Immune infiltration analysis and gene set enrichment analysis (GSEA) was used to evaluate the immune cell context and the enriched gene sets among the subgroups.

**Results:** Compared with adjacent normal tissue, differentially expressed 24 m6A regulators were identified in breast cancer. According to the expression features of m6A regulators above, we established two subgroups of breast cancer, which were also surprisingly distinguished by the feature of the immune microenvironment. The Model based on modification patterns of m6A regulators could predict the patient’s T stage and evaluate their prognosis. Besides, the low m6aRiskscore group presents an immune-activated phenotype as well as a lower tumor mutation load, and its 5-years survival rate was 90.5%, while that of the high m6ariskscore group was only 74.1%. Finally, the cohort confirmed that age (*p* < 0.001) and m6aRiskscore (*p* < 0.001) are both risk factors for breast cancer in the multivariate regression.

**Conclusion:** The m6A regulators play an important role in the regulation of breast tumor immune microenvironment and is helpful to provide guidance for clinical immunotherapy.

## Highlight

1) The heterogeneity of breast cancer is revealed by the classification based on m6A
2) The risks of breast cancer patients are significantly different between subtypes identified by m6A regulators
3) Molecular subtypes of breast cancer that respond to immunotherapy have been identified, providing impetus for breast cancer immunotherapy

## Introduction

As a highly heterogeneous with complex histological characteristics and genetic features, breast cancer ranks first cancer in women on a global scale, and immunotherapy is currently a promising therapeutic strategy for advanced breast cancer (Emens, 2018). Therefore, exploring the molecular mechanism of breast cancer tumorigenesis and development is crucial to advance clinical diagnosis.

As the most widely distributed internal modification form of mRNA in eukaryotic cells, m6A modification participates in the occurrence and development of human cancer. Increasing researchers have come to demonstrate that dysfunction in m6A modification could affect the phenotype of tumor cells in breast cancer, colorectal cancer, and other cancers (Deng et al., 2019; Niu et al., 2019; Yang et al., 2020). He Chuan et al. reported that YTHDF3 induces the translation of m6A-Enriched key brain metastatic genes to promote BC brain metastasis (Chang et al., 2020). As previously described, m6A modification is a dynamic and reversible process (Yang et al., 2018). This modification in cells starts with methyltransferases (“writers”). Its regulatory factors include METTL3/14/16, WTAP, RBM15, RBM15B, ZC3H13, CBLL1, and KIAA1429; reversing m6A modification is mediated by demethylases (“erasers”), including the notorious obesity-associated protein (FTO) and ALKBH5. The RNA information modified by m6A regulators needs to be recognized by the RNA binding protein (“readers”), composed of LRPPRC, HNRNPA2B1, FMR1, IGF2BP1/2/3, HNRNPC, ELAVL1, YTHDC1/2, and YTHDF1/2/3 so as to participate in downstream RNA translation and degradation processes (Yang et al., 2018; He et al., 2019).

Recently, some studies have reported a special connection between m6A regulators and the tumor microenvironment (TME) (Tang et al., 2020; Zhang et al., 2020). Due to the immune cell-tumor cell interactions, various biological behaviors such as immune escape and immune tolerance contribute to tumorigenesis. Han et al. have identified YTHDF1 as an immune suppressor in tumors (Han et al., 2019). Inhibiting the expression of YTHDF1 in classical dendritic cells can enhance the cross-presentation of tumor antigens and the cross-activation of CD8^+^ T cells *in vivo* (Han et al., 2019). Whereas another m6A regulator, METTLE3, can activate T cells by enhancing translation of CD80, CD40, and TLR4 signaling adaptor Tirap in dendritic cells. Li et al. have reported that METTL3-deficient T cells lose the ability to produce specialized immune cells (Li et al., 2017). These findings reveal the possible protective effect of m6A (Wang H. et al., 2019). Hence it is speculated that m6A modification may affect the occurrence and development of tumors by regulating the characteristics of TME.

More specifically, breast cancer, especially triple-negative breast cancer (TNBC), which has a relatively low immune response after immunotherapy, has always been considered as “cold tumor” (Bianchini et al., 2016). But increasing studies have observed various immune infiltrations in TME of BC (Gil Del Alcazar et al., 2020). Therefore, the selection of appropriate subtypes is crucial to the effectiveness of immunotherapy.

**FIGURE 1:**
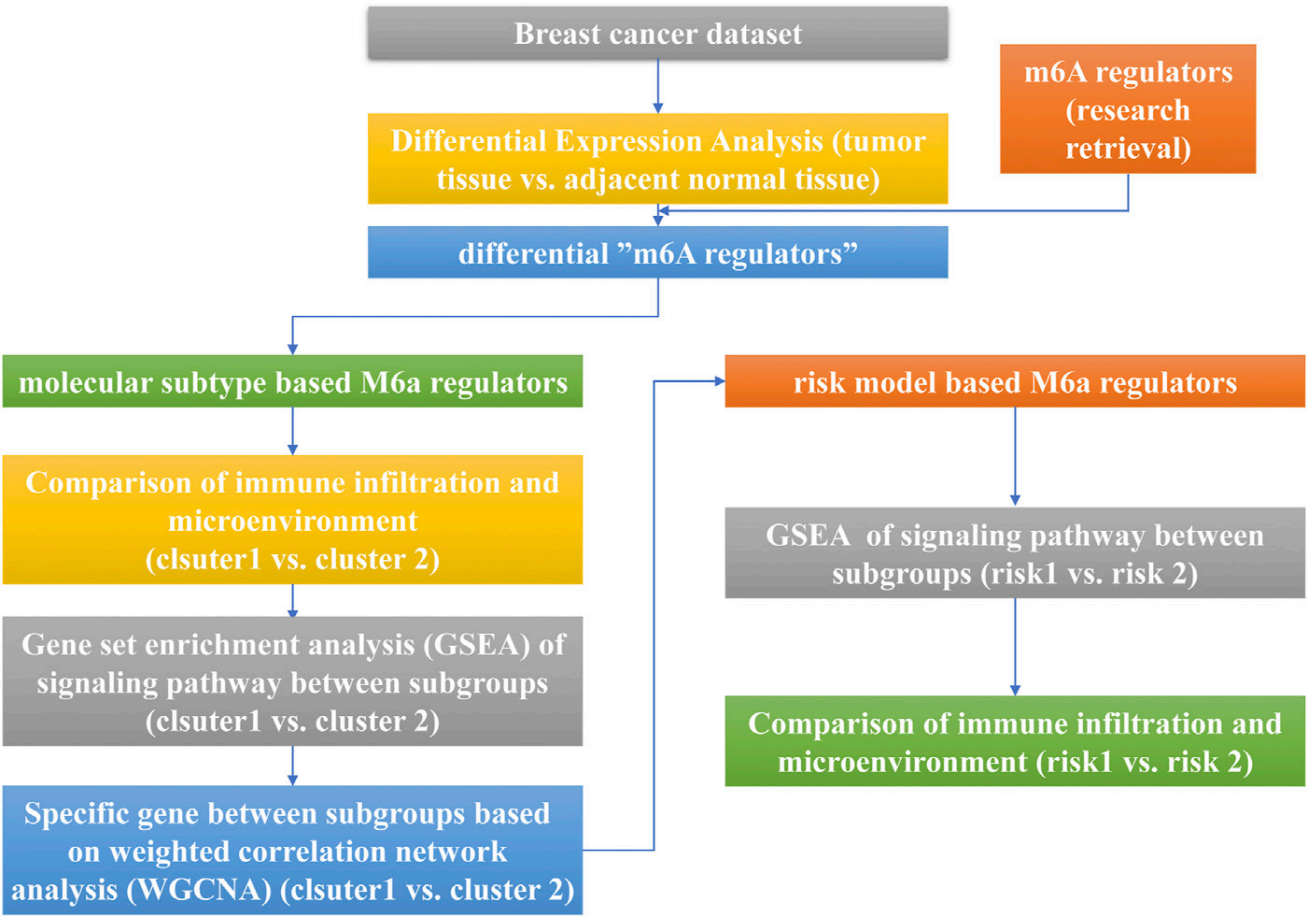
The flow chart of the study design and analysis.

In this work, we integrated the genomic information of breast cancer samples in the TCGA database to evaluate the breast cancer m6A modification patterns comprehensively and to explore the features of immune cell infiltration under distinct m6A modification modes (Figure 1). We found that there are two different m6A modification patterns in breast cancer patients, and significant distinctions were presented in the immune infiltration characteristics of the two types of patients. Therefore, we established a risk scoring model based on m6A modification patterns to predict the overall survival rate and provide guidance for clinical treatment classification.

## Material and Methods

### Breast Cancer Data Sources

In this study, transcriptome dataset and corresponding of 1222 TCGA-BRCA (The Cancer Genome Atlas-Breast Cancer) samples and GSE86374 cohort (containing 159 samples, GEO databases) were included in the present study. We excluded the patients without survival information from further analysis. The clinical information of 893 patients was summarized in Table 1.

**TABLE 1:** Clinicopathological features of patients included in this study annotated from TCGA database.

	Level	Overall	High-m6aRiskscore	Low-m6aRiskscore	p-value
number	Alive	893	435	458	0.002
fustat (%)		769 (86.1)	358 (82.3)	411 (89.7)	
	Dead	124 (13.9)	77 (17.7)	47 (10.3)	0.317
age (%)	≤65	663 (74.2)	330 (75.9)	333 (72.7)	
	>65	230 (25.8)	105 (24.1)	125 (27.3)	
gender (%)	FEMALE	882 (98.8)	430 (98.9)	452 (98.7)	1
	MALE	11 (1.2)	5 (1.1)	6 (1.3)	
stage (%)	Stage I	159 (17.8)	87 (20.0)	72 (15.7)	0.122
	Stage II	521 (58.3)	238 (54.7)	283 (61.8)	
	Stage III	196 (21.9)	103 (23.7)	93 (20.3)	
	Stage IV	17 (1.9)	7 (1.6)	10 (2.2)	
T (%)	T1	235 (26.3)	125 (28.7)	110 (24.0)	0.014
	T2	529 (59.2)	235 (54.0)	294 (64.2)	
	T3	98 (11.0)	58 (13.3)	40 (8.7)	
	T4	31 (3.5)	17 (3.9)	14 (3.1)	
M (%)	M0	876 (98.1)	428 (98.4)	448 (97.8)	0.702
	M1	17 (1.9)	7 (1.6)	10 (2.2)	
N (%)	N0	438 (49.0)	208 (47.8)	230 (50.2)	0.885
	N1	299 (33.5)	151 (34.7)	148 (32.3)	
	N2	103 (11.5)	50 (11.5)	53 (11.6)	
	N3	53 (5.9)	26 (6.0)	27 (5.9)	

The data set in TCGA uses the R package TCGAbiolinks to download all gene expression RNA sequencing data from Genomic Data Commons (GDC, https://portal.gdc.cancer.gov/). Somatic mutation data were obtained from the TCGA database. The data were analyzed using R (version 4.0.2) and R Bioconductor packages.

### Select m6A Regulator and Unsupervised Analysis Using ConsensusClusterPlus Methods

According to the published studies (Zhang et al., 2020), we selected 24 m6A regulators for unsupervised cluster analysis. These 24 m6A regulators include 9 writers (METTL3/14/16 and WTAP, RBM15, RBM15B, ZC3H13, KIAA1429, and CBLL1), 2 erasers (FTO, ALKBH5), and 13 readers (YTHDF1/2/3, YTHDC1/2, HNRNPA2B1, HNRNPC, LRPPRC, FMR1, ELAVL1, and IGF2BP1/2/3). With the “limma” package (http://www.bioconductor.org/packages/release/bioc/html/limma.html), differentially expressed of the m6A regulators in BC was unveiled between tumor tissues and normal tissues. The number of clusters is determined by the algorithm of unsupervised cluster analysis, performed with the R package “ConsensuClusterPlus” (50 iterations, 80% resampling rate, Pearson correlation) (https://www.bioconductor.org/packages/release/bioc/html/ConsensusClusterPlus.html). We also performed principal component analysis (PAC) on the clustering results using the R package “PCA” to study gene expression patterns in breast tumor clusters. The PPI network was built using the Retrieval of Interacting Genes (STRING, http://string.embl.de/).

### Gene Set Enrichment Analysis and Analysis of Immune Cell Infiltration

Enrichment of functions and signaling pathways of different clusters were evaluated in software GSEA 4.1.0. In order to investigate the TME of breast cancer, we used the CIBERSORT analysis tool to calculate the number and type of immune cells infiltrated by each sample (Newman et al., 2015). The CIBERSORT score can be found in The Cancer Immunome Atlas (TCIA, https ://tcia.at/, created by Pornpimol et al.). The level of immune cell infiltration of each patient in TCGA-BRCA cohort has also been collected from the Tumor immune estimation resources (TIMER) platform (https://cistrome.shinyapps.io/timer/).

### Hub Genes in the Module of Interest Based on Weighted Correlation Network Analysis

“Limma” package was employed to extract all the differential genes (DEGs) in the expression matrix of the GSE86374 cohort for WGCNA. The R package “WGCNA” was used to find the modules and hub genes related to the m6A subgroup (Langfelder and Horvath, 2008). We convert the adjacency matrix into a topological overlap matrix (TOM), and genes are divided into different gene modules based on the TOM diversity measure. Here, the soft threshold power is set to 9 (scale-free R2 = 0.85); the cutting height is set to 20,000, and the minimum module size is set to 10 to identify key modules. The hub gene is defined as a gene with module membership (MM) > 0.8 and gene significance (GS) >0.85.

### Verify the Prognostic Value of the Hub Gene

In order to screen out genes with clinical predictive value in the hub gene, the LASSO Cox regression algorithm was applied to the hub gene in the GSE86374 cohort (Ramsay et al., 2018). 14 genes were selected according to the minimum standard to construct the risk profile, and the coefficient obtained from the LASSO algorithm was used to calculate the risk score of each patient, as shown below:

$$m6\mathrm{aRiskscore}=\overset{n}{\underset{i=1}{\sum }}\exp\left(i\right)\mathrm{*\theta}\left(i\right)$$

Where n is the number of prognostic genes, exp(i) is the expression level of gene i, and α(i) is the regression coefficient of gene i in the LASSO algorithm. According to the average risk score, it will be divided into a high-m6aRiskscore group and a low-m6aRiskscore group. We used the R package “survival” to evaluate the difference in survival time between the two groups. Finally, the “PROC” R package was used to quantify the area under the curve (AUC) to measure the specificity and sensitivity of m6aRiskscore.

### Statistical Analysis

We employed the Kaplan-Meier method to generate a survival curve for prognostic analysis, and the log-rank test for comparison. Spearman and Pearson’s correlation analysis was used to calculate the correlation between m6aRiskscore and TME infiltrating immune cells or between m6aRiskscore and the expression of m6A regulators, respectively. Tumor mutation burden (TMB) was calculated from the number of non-synonymous alterations for each patient (Cancer Genome Atlas Research Network, 2012). The t-test is used to investigate the difference in risk distribution among clinical groups. The single factor and multivariate regression are used to determine the factors that affect prognosis in the TCGA-BRCA cohort, and we use the “forestplot” R package to visualize the results. The waterfall function of the “Maftools” package (https://bioconductor.org/packages/release/bioc/html/maftools.html) was used to display the mutation status of patients in the TCGA-BRCA cohort. TIDE algorithm was used to predict potential immune checkpoint blockade response (Jiang et al., 2018). All statistical results with *p* < 0.05 were considered statistically significant.

## Results

### Genetic Variation of 24 m6A Regulators and its Clinicopathological Association

**FIGURE 2:**
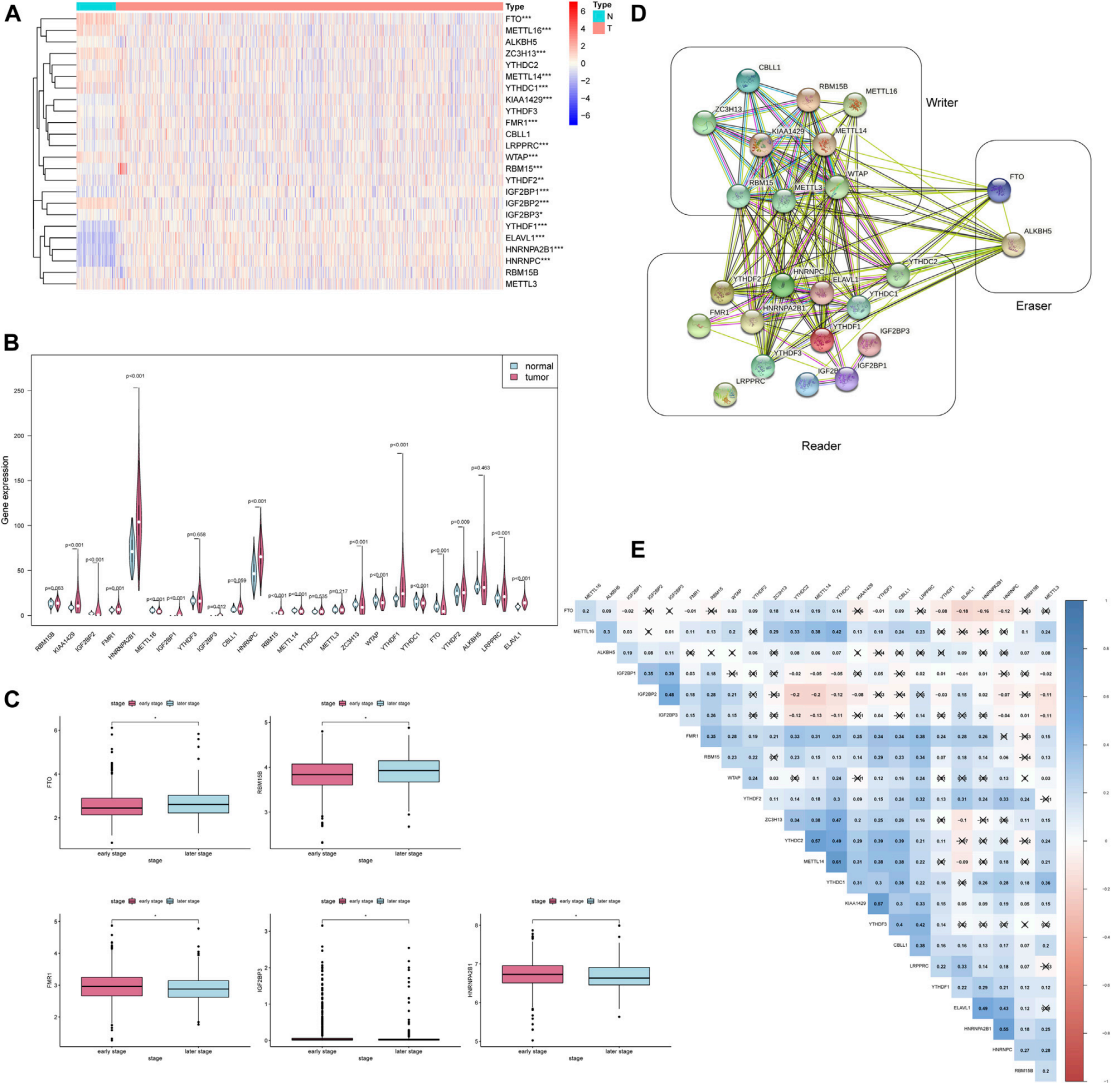
The expression of 24 m6A regulators is associated with clinicopathological characteristics. * * *p < 0.001, * *p < 0.01, *p < 0.05. **(A)** The heatmap of 24 m6A regulators in different tumor tissues. **(B)** The violin plot of 24 m6A regulators in different tumor tissues. **(C)** The boxplot of 5 m6A regulators in the different pathological stages. **(D)** The PPI network of the 24 m6A regulators constructed using STRING. **(E)** Spearman correlation analysis of 24 m6A regulators.

**FIGURE 3:**
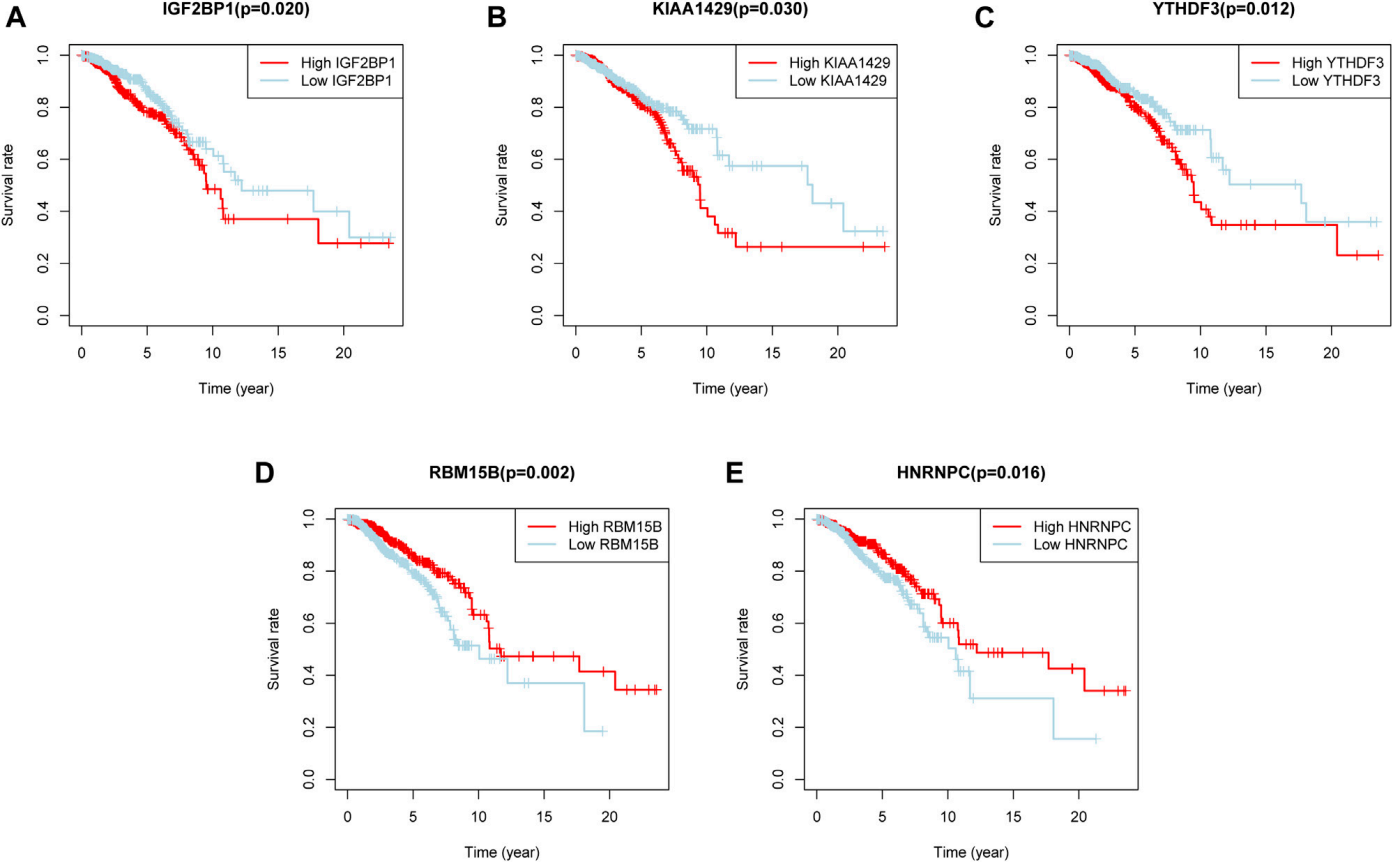
Five m6A regulators were associated with overall survival. **(A–E)** The overall survival curves of m6A regulators. *p* < 0.05 were considered statistical significance.

To find out m6A modification characteristics in breast cancer, we explored the expression level of 24 regulators in different sample tissues, including tumor status (normal and tumor) and pathological stage (early stage covering Stage I and II, later stage covering Stage III and IV). It appeared that IGF2BP1/3, ELAVL1, HNRNPA2B1, HNRNPC, KIAA1429, RBM15, LRPPRC, FMR1, YTHDF1/2 are highly expressed in the tumor while FTO, METTL14/16, WTAP, YTHDC1 and ZC3H13 are down-regulated (Figures 2A,B). And the five of them (FTO, RBM15B, FMR1, IGF2BP3, and HNRNPA2B1) are also closely related to their staging (Figure 2C). Survival analysis further confirmed the relationship between m6A regulators and prognosis. IGF2BP1, KIAA1429, and YTHDF3 are associated with poor overall survival (OS), while high RBM15B and HNRNPC indicate a better prognosis (Figures 3A–E).

**FIGURE 4:**
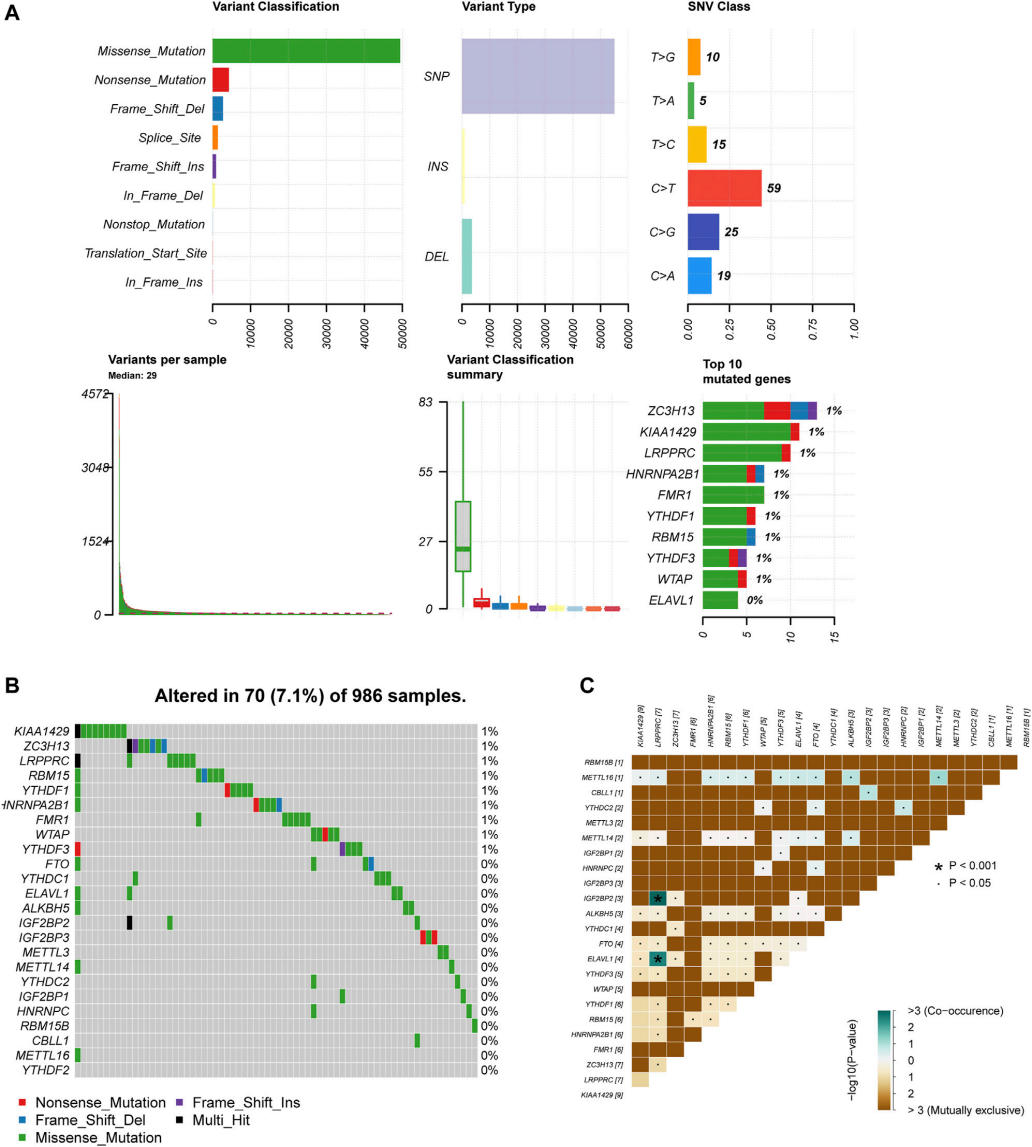
M6A regulators present a low genetic variation rate. **(A)** The summary of 24 m6A regulators copy number variation and somatic mutations in breast cancer. **(B)** The mutation frequency of 24 m6A regulators 986 samples. **(C)** Co-mutation among 24 m6A regulators.

In order to determine whether the above-mentioned m6A regulators’ expression alteration is caused by mutations, we first summarized the incidence of 24 m6A regulators’ copy number variation and somatic mutations in breast cancer (Figure 4A). But out of 986 samples, only 70 of them had mutations with a 7.1% mutation frequency (Figure 4B). It indicates that somatic mutation may not be the main reason for the alteration of m6A regulators. Consistent with this result, the study of co-mutation reveals that only ELAVL1, IGF2BP2 and LRPPRC showed a significant co-mutation relationship (Figure 4C). Their co-mutation may be explained by chromosomal translocation. It is known that ELAVL1 is located on chromosome 19p13.2, LRPPRC on chromosome 2p21, and IGFBP2 on chromosome 3q27.2. Dowiak and Tirado have reported the chromosomal aberrations of t(2;3) (p13-25;q25-29) in myeloma, which provided us with hints for explaining co-mutations (Dowiak and Tirado, 2017).

To further explore whether these 24 m6A regulators have any meaningful interactions, we established a PPI network and Spearman correlation analysis. It can be observed that apart from LRPPRC, the other 23 m6A regulators possess a complex network of interactions, and most of them are positively correlated, especially the correlation between METL14 and YTHDC1 reached 0.61 (Figure 2E). The PPI diagram points out that there is a close connection between the 9 writers (Figure 2D); compared to the strong connection between the reader and the writer, the 13 readers have relatively few connections among themselves (Figure 2D). Since the effect of methylation on the stability of the transcript mainly depends on which reader is dominant in the cellular environment, this feature of reader may help cells to perform specific functions.

### Consensus Clustering of 24 m6A Regulators Identified Two Clusters of Patients With Different TME Cell Infiltration Characteristics

**FIGURE 5:**
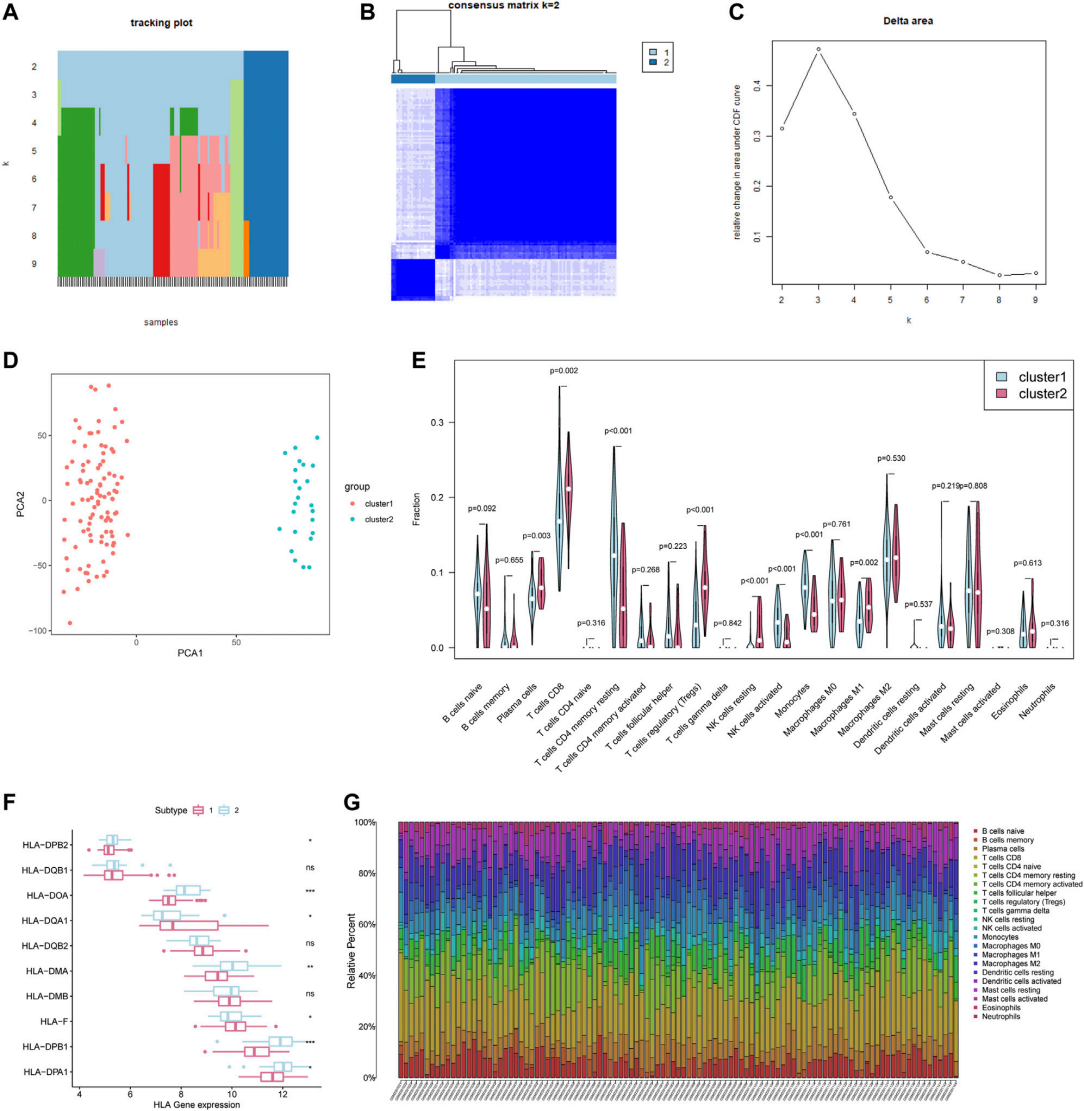
Consensus Clustering of 24 m6A regulators identified two clusters of patients with different TME cell infiltration characteristics. **(A)** The tracking plot for k = 2 to k = 9. **(B)** The heatmap for k = 2. **(C)** Relative change in area under CDF curve for k = 2–9. **(D)** Principal component analysis of the total RNA expression profile. **(E)** Different immune cell content in tumor tissue. The green represents Cluster1 and the red represents Cluster2. **(F)** HLA gene expression in cluster1/2. **(G)** Relative Percent of different immune cells in each sample.

According to the expression of 24 m6A regulators, we used the “ConsensusClusterPlus” R package to classify patients. We observed that when k = 2, the interference between subgroups is relatively smaller (clustering increasing from k = 2–9). And to facilitate subsequent analysis, two subgroups were distinguished (Figures 5A–C). Among them, cluster1 contains 99 patients and cluster2 contains 24 patients. PCA proved the difference between the two subgroups (Figure 5D). Next, we conducted a GSEA analysis to explore the biological behavior between these two different subgroups (Supplementary Figures S1A–E). GSEA analysis showed that Cluster1 exhibited active protein transcription and post-translational modification processes, including up-regulation of mRNA metabolic process (NES = 1.75, normalized *p* = 0.012), mRNA transport (NES = 1.69, normalized *p* = 0.036), and N terminal protein amino acid modification pathways (NES = 1.86, normalized *p* = 0.010). Cluster2 is enriched in apoptosis-related pathways, including regulation of mitochondrial membrane permeability in apoptotic process (NES = −1.60, normalized *p* = 0.015) and autophagosome membrane (NES = −1.62, normalized *p* = 0.030).

We further analyzed the differences in the infiltration of immune cells between the two subgroups. The differences lie mainly in plasma cells, CD8^+^ T cells, CD4^+^ T cells memory resting, T cells regulatory (Tregs), NK cells, Monocytes, Macrophages M1 (Figure 5E). The plasma cells and CD8^+^ T cells of Cluster1 were both significantly lower than those of Cluster2, but the activated Monocytes and NK cells were higher than those of Cluster2 (Figure 5E). Meanwhile, the antigen-processing machinery (APM) component HLA-II genes in cluster2 present a higher expression level (Figure 5F). The previous research referred to the subgroups with this immune characteristic as immune activation phenotype (characterized by immune activation and adaptive immune cell infiltration) and immune rejection phenotype (characterized by innate immune cell infiltration) (Zhang et al., 2020). What is worth noting is that the number of CD8 + cells, the major labor of tumor removal, account for nearly 25% of the total number of cells in breast cancer (Figure 5G), is lower in cluster1 (*p* = 0.002). Additionally, some important immune activation-related genes such as TNF, CD8A, and CXCL9 are present at a lower level in cluster1, with a higher expression of immune checkpoint PDCD1 (Supplementary Figures S2A–E). In summary, we can speculate that the adaptive immune response of Cluster1 may be less activated than Cluster2.

### WGCNA and a Risk Signature Established Based on Hub Gene

**FIGURE 6:**
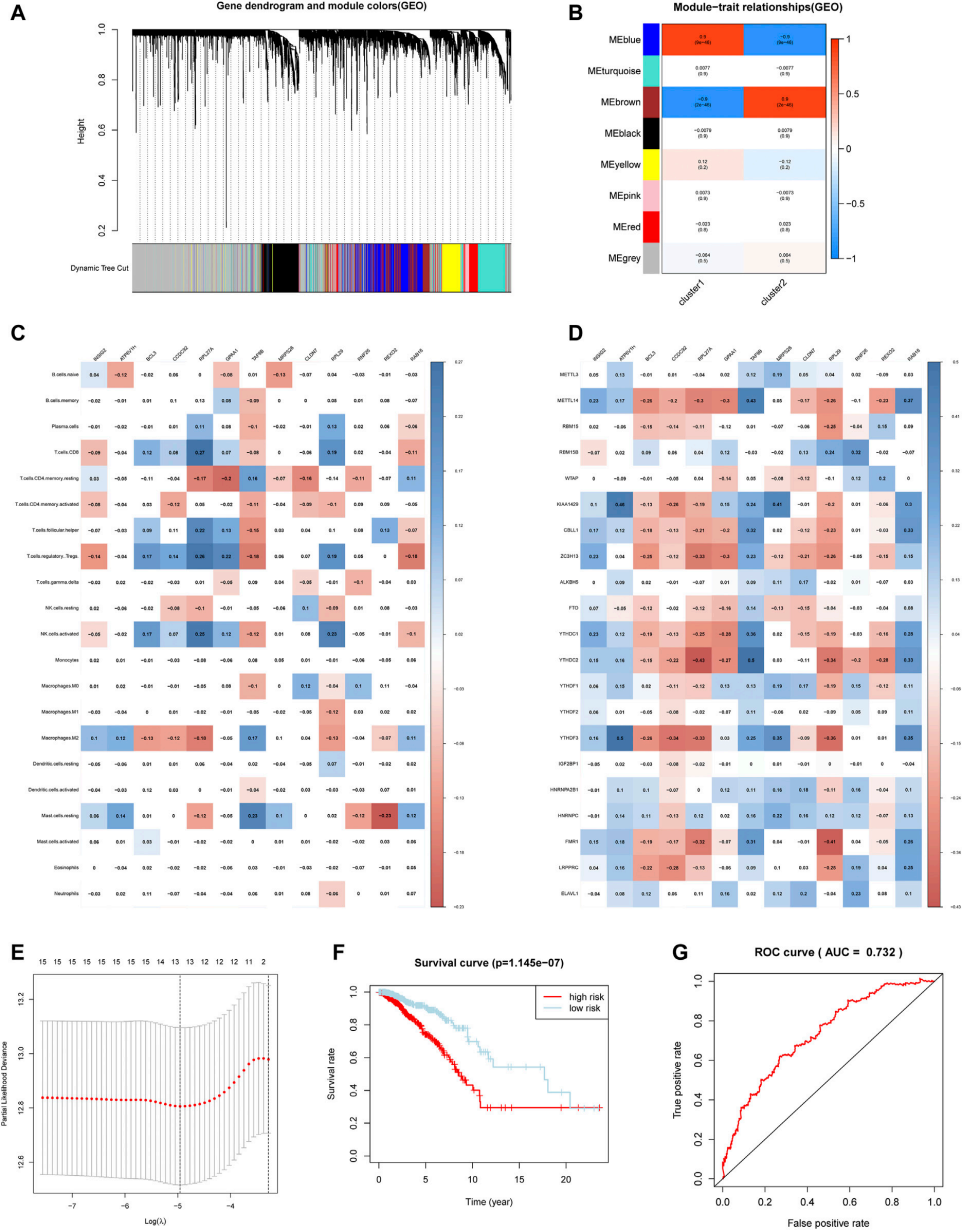
Hub genes were significantly correlated with m6A regulators and immune infiltrating cells. **(A)** Clustering dendrograms of genes. **(B)** Module-trait associations. **(C)** Spearman correlation analysis between hub gene and 24 m6A regulators. **(D)** Spearman correlation analysis between hub gene and infiltrating immune cells. **(E)** The minimum mean value of the target parameter in the process of LASSO Cox regression. **(F)** The overall survival of the high and low m6aRiskscore groups. **(G)** The ROC curve verifies the accuracy of the risk model.

In order to find the key genes that are most related to the m6A regulators in breast cancer, we identified 8 modules for setting the soft threshold power and cutting height (Figures 6A,B). According to the module correlation heat map, it is found that the brown module and the blue module have the highest correlation (Figure 6B).

Then we applied limma analysis to extract all the differential genes of the two subgroups. These 653 DEGs are respectively intersected with the brown module and the blue module, and the union of the two sets of results formed a hub gene set. Figure 6C showed that the expression of the selected hub gene was significantly correlated with m6A regulators, among which BCL3, CCDC92, RPL27A, and RPL29 were positively correlated with most m6A regulators. Aside by this, the expression of the hub gene is also associated with some of the immune infiltrating cells. Consistent with the previous differences in immune cell infiltration in the m6A regulator subgroup, most of the hub genes are related to CD4^+^ T cells memory resting, CD8^+^ T cells, T cells regulatory (Tregs), and NK cells activated (Figure 6D).

Considering the complexity and heterogeneity of m6A individual modification, we constructed a scoring system to quantify the risk of a single breast cancer patient by applying the LASSO Cox regression algorithm and the minimum absolute contraction (Figure 6E). The scoring system divides patients into high-m6aRiskscore and low-m6aRiskscore groups, 534 and 535 patients were included respectively. Firstly, Kruskal-Wallis test illustrated that patients with lower m6aRiskscore present a higher 5-years survival rate, and the 5-years survival rates of the high and low m6aRiskscore groups were 74.1 and 90.5%, respectively (Figure 6F). The ROC curve verifies the accuracy of the model (AUC = 0.732) (Figure 6G). Then the GSEA analysis indicates that the low-m6aRiskscore group was enriched in immune response-related pathways such as complement and coagulation cascades (NES = 1.95, normalized *p* = 0.004), intestinal immune network for IgA production (NES = 1.73, normalized *p* = 0.025) (Supplemetary Figures S3A–B); the high-m6aRiskscore group was enriched with many malignant hallmarks of cancer, including ubiquitin-mediated proteolysis (NES = −1.84, normalized *p* = 0.004), mismatch repair (NES = −1.81, normalized *p* = 0.004), glycosylphosphatidylinositol GPI anchor biosynthesis (NES = −1.78, normalized *p* = 0.010) and cell cycle (NES = −1.73, normalized *p* = 0.025) (Supplemetary Figures S3C–F). The above results suggest that the m6aRiskscore based on m6A regulators is closely related to the malignancy and immunological competence of breast cancer, and thus it can be an indicator of the prognosis and treatment of breast cancer.

### Prognostic Risk Score Indicated Strong Associations With Pathological Stage and Tumor Somatic Mutation in Breast Cancer

**FIGURE 7:**
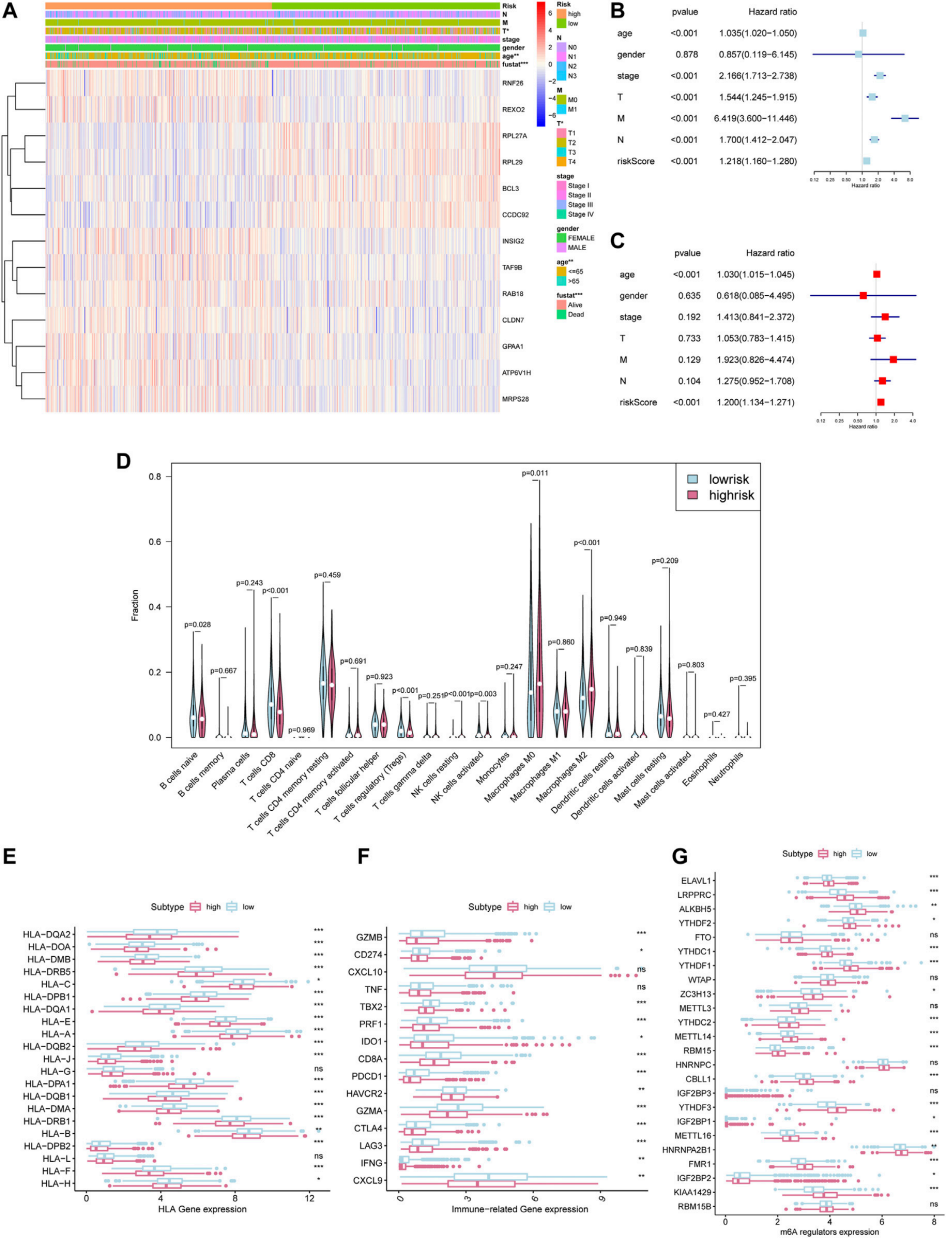
Prognostic risk score indicated strong associations with pathological stage and tumor somatic mutation in breast cancer. *p < 0.05, **p < 0.01 and ***p < 0.001. **(A)**The heatmap of 24 m6A regulators in high-m6aRiskscore and low-m6aRiskscore breast cancer. **(B)** Univariate Cox regression analysis to estimate the prognostic significance of clinicopathological factors and m6aRiskscore. **(C)** Multivariate Cox regression analysis to estimate the prognostic significance of clinicopathological factors and m6aRiskscore. **(D)** Different immune cell content in tumor tissue. The green represents the low-m6aRiskscore group and the red represents the high-m6aRiskscore group. **(E)** The expression level of HLA-related gene in the subgroups. **(F)** The expression level of immune-related gene in the subgroups. **(G)** The expression level of m6A regulators in the subgroups.

The heat map shows the expression level of the selected hub gene and its relationship with clinical characteristics (Figure 7A). The subgroups divided by m6aRiskscore are related to the T stage (*p* < 0.05), age (*p* < 0.01), and survival status (*p* < 0.001). Afterwards, Univariate and multivariate Cox regression further explored whether risk characteristics are an independent prognostic factor. The results demonstrated that in single-factor analysis, age, pathological stage, stage TNM, and m6aRiskscore are all risk factors (Figure 7B), while in multivariate analysis, only age and m6aRiskscore are still risk factors (*p* < 0.001) (Figure 7C), which proves that m6aRiskscore can be independent of breast cancer prognostic biomarkers.

**FIGURE 8:**
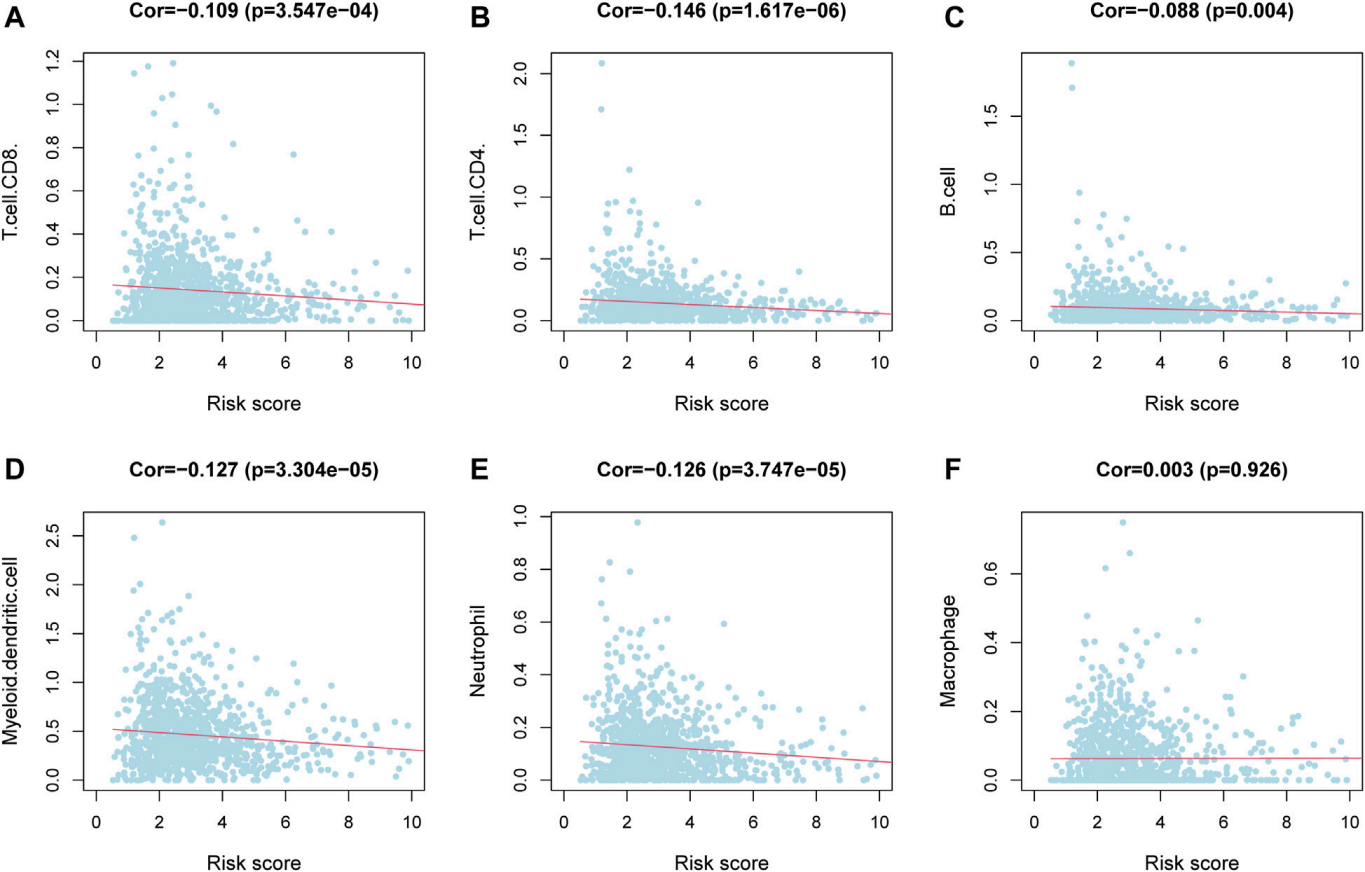
Correlation between immune cells and m6aRiskscore was analyzed using the TIMER database. **(A–F)** Correlation analysis between immune cells and m6aRiskscore in TCGA-BRCA cohort.

To clarify the correlation between the m6aRiskscore and the host anti-tumor immune response, we performed immune infiltration analysis on the subgroup divided by m6aRiskscore (Figure 7D). CD8^+^ T cells, Naïve B cells, and NK cells activated were all lower in the high-m6aRiskscore group than in the low-m6aRiskscore group. Besides, macrophages M2, M0, and NK cells resting present higher expression than the low-m6aRiskscore group. Similarly, in the correlation analysis of immune cells using the TIMER database, it was found that B cells (cor = -0.088), CD4^+^ T cells (cor = -0.146), CD8^+^ T cells (cor = -0.109), dendritic cells (cor = -0.127) and neutrophils (cor = -0.126) were all negatively correlated with m6aRiskscore (Figures 8A–F).

Consistent with the characteristics of immune infiltration, the expression of almost all MHC molecules, including MHC1 and MHC2, was lower in the high-m6aRiskscore group (Figure 7E). Given that the antigen deficiency of MHC molecules is closely related to tumor escape, this result indicates a weaker tumor immunogenicity of the high-m6aRiskscore group. In terms of immunoregulatory genes, the low-m6aRiskscore group showed higher levels of immune-activating genes, such as TBX2, CD8A, CXCL9, GZMA, GZMB, PRF1, and IFNG (Figure 7F). But it is interesting that the immune checkpoints CD274 and PDCD1 in the low-m6aRiskscore group are also lower (Figure 7F). Moreover, expression levels of m6A regulators between the two groups were also investigated. We surprisingly found that most of the m6A regulators are higher in a high-risk group, which is worthy of discussion (Figure 7G). To sum up, combined with the immune infiltration landscapes like high M2 cells, low CD8^+^ cells, and low MHC molecules in the high-m6aRiskscore group, we tend to believe that the high-m6aRiskscore group exhibits immunosuppressive characteristics, which may be one of the reasons for its poor prognosis (Figure 6F).

### M6ariskscore Predicts the Response of Immunotherapy

**FIGURE 9:**
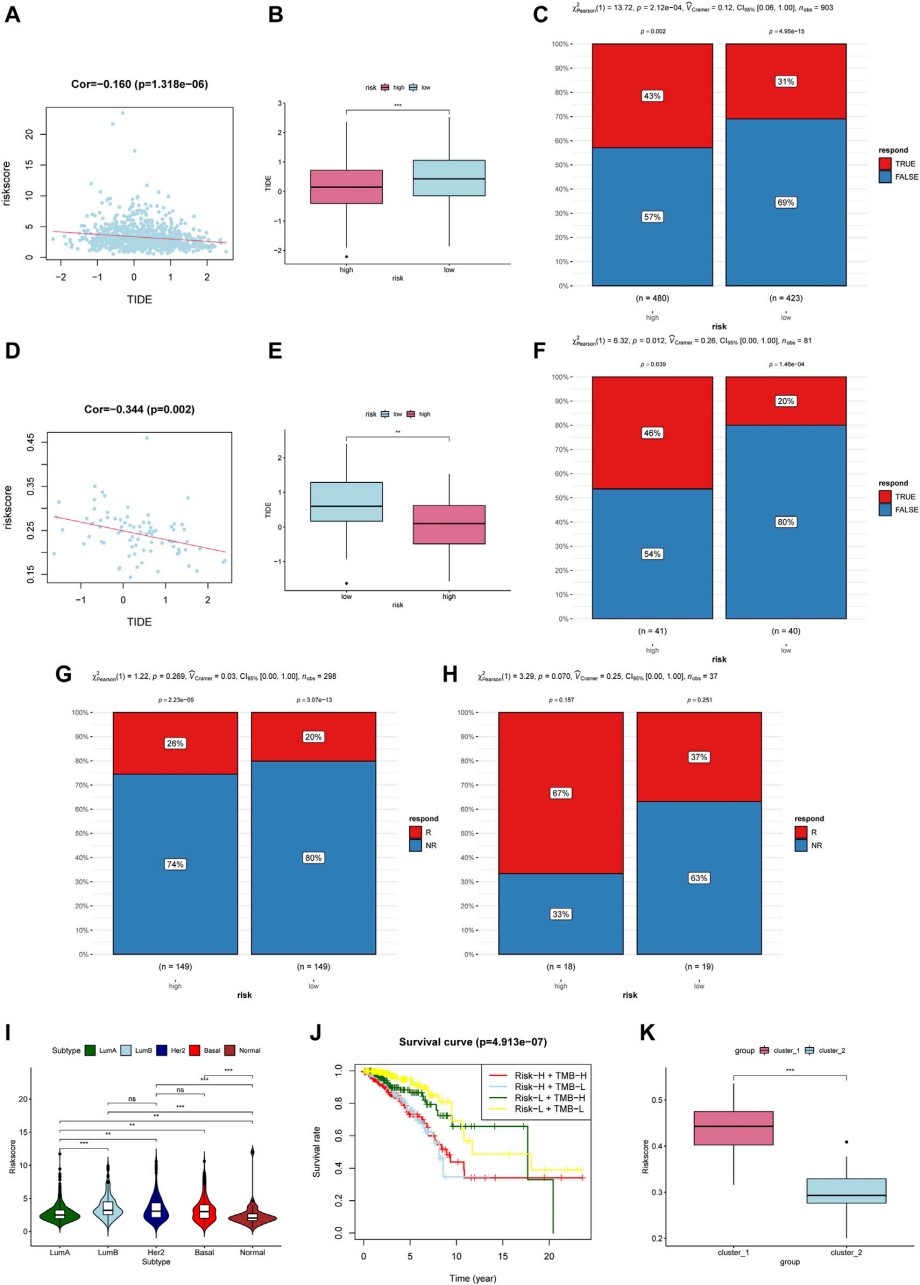
M6ariskscore predicts the response of immunotherapy. **(A)** Correlation analysis between TIDE value and m6aRiskscore in the TCGA-BRCA cohort. **(B)** Boxplot of TIDE value between subgroups in the TCGA-BRCA cohort. **(C)** Rate of clinical response estimated by TIDE in high or low m6aRiskscore groups in the TCGA-BRCA cohort. **(D)** Correlation analysis between TIDE value and m6aRiskscore in the GSE48391 cohort. **(E)** Boxplot of TIDE value between subgroups in the GSE48391 cohort. **(F)** Rate of clinical response estimated by TIDE in high or low m6aRiskscore groups in the GSE48391 cohort. **(G)** Rate of clinical response (response [R]/no response [NR]) to anti-PD-L1 immunotherapy in high or low m6aRiskscore groups in the IMvigor210 cohort. **(H)** Rate of clinical response (R/NR) to immunosuppressives (methotrexate and cyclophosphamide) in high or low m6aRiskscore groups in the GSE42664 cohort. **(I)** m6aRiskscore in different molecular subtype of breast cancer. **(J)** Survival analyses for patients stratified by both TMB and m6aRiskscore. **(K)** Boxplot of m6aRiskscore between Cluster1 and Cluster2.

Due to the lack of breast cancer immunotherapy cohort, we use Tumor Immune Dysfunction and Exclusion (TIDE) to predict the response of patients with immune checkpoint blockade (ICB). TIDE is an algorithm for predicting ICB response based on gene expression profiles, and a low TIDE score indicates that it is more likely to become a responder for immunotherapy (Jiang et al., 2018). Recent studies have proven that TIDE scores demonstrate higher prediction accuracy than PD-L1 expression levels and TMB (Jiang et al., 2018; Wang S. et al., 2019; Keenan et al., 2019). In our study, the TIDE score was negatively correlated with m6aRiskscore in the TCGA-BRCA cohort and external datasets GSE48391 (Figures 9A,D). And the TIDE score of the high-m6aRiskscore group is significantly lower than the low-m6aRiskscore group (Figures 9B, E), which means that the high-m6aRiskscore group is more likely to benefit from immunotherapy (Figures 9C,F).

We further validated the effectiveness in other cancer cohorts involving immunotherapy. Although a significant difference was not observed statistically, all the high-m6aRiskscore groups have a higher percentage to benefit from immunotherapy. As shown in Figures 9G,H below, the response rate of anti-PD-L1 therapy in the high-m6aRiskscore group (26%) was higher than that in the low-m6aRiskscore group (20%) in urothelial carcinoma (Figure 9G). Similarly, the response rate of immunosuppressives in the high-m6aRiskscore group (67%) was also higher than that in the low-m6aRiskscore group (37%) in large granular lymphocyte leukemia (Figure 9H). The lack of significant statistical differences in our results may be due to different types of cancer. Moreover, A study using TIDE to evaluate the response of ICB in lung adenocarcinoma also showed that high-risk groups with immunosuppressive phenotypes are more likely to benefit from immunotherapy, which is consistent with our conclusion (Wang et al., 2020b). These data indicate that the m6aRiskscore may be related to the response to immunotherapy.

Further, we examined the correlation between m6aRiskscore and molecular subtypes. Figure 9I shows that high-m6aRiskscore is related to Luminal B, HER2-enriched, and TNBC, while the m6aRiskscore of Luminal A and Normal-like BC is relatively low. Next, we calculated the tumor mutation burden (TMB) of each patient. Patients with lower tumor neoantigen load and lower m6aRiskscore have the highest 5-year survival rate (Figure 9J). In the end, we compared the subgroups based on m6A regulators and the subgroups based on m6ariskscore. The two ways of classification showed consistency given that Cluster1 had higher m6aRiskscore (Figure 9K), which is consistent with previously identified immunosuppressive phenotypes.

## Discussion

In this context, we aimed to determine the prognostic value of m6a-related mRNA in breast cancer and its role in the tumor microenvironment. For this reason, we clustered the GEO cohort into two subgroups. The two subgroups have significant differences in malignant tumor markers, immune cell infiltration, and expression of immune regulatory genes. Next, based on the differential hub genes of the two subgroups, we develop a new biomarker risk prediction model. The results based on 1069 TCGA-BRCA samples show that m6aRiskscore helps to identify patients with high immunogenicity, and is significantly related to the m6a modification characteristics, immune microenvironment, molecular subtypes, and patient prognosis of breast cancer.

At present, researchers are beginning to uncover the mystery of m6a shaping the tumor immune microenvironment (Han et al., 2019; Wang et al., 2020a), A review has comprehensively summarized the involvement of m6A in innate and adaptive immune cell regulation (Ma et al., 2021). In our research, we discovered that KIAA1429 is highly expressed in tumor tissues compared with adjacent tissues and higher KIAA1429 means worse survival time. Following previous studies, KIAA1429 plays a role as a carcinogen of breast cancer (Qian et al., 2019). Lan T also reported that in hepatocellular carcinoma KIAA1429 controls the differentiation of T helper 2 (Th2) by inducing separation of RNA binding protein HuR, and so affects the expression of IL-4, IL-5, and IL-13 (Lan et al., 2019). Therefore, KIAA1429 may play a prominent role in tumor immune regulation. In addition, the expression level of YTHDFs in tumor tissues is also significantly different from normal tissues, and among them YTHDF3 is negatively correlates with survival rate, which is consistent with previous studies (Anita et al., 2020; Chang et al., 2020). Han and Liu also pointed out that YTHDF1 can promote the transcription of lysosomal cathepsin and therefore inhibit the antigenic crossover of classical dendritic cells (Han et al., 2019). Besides, m6A regulators such as METTL14 have been reported to be involved in the regulation of T cell homeostasis (Han et al., 2019). These m6A regulators, which play an important role in the occurrence and development of breast cancer, also showed generally a higher expression in the high-m6aRiskscore group, prompting us to assume m6A regulators are closely involved in the regulation of the breast cancer immune microenvironment.

In April 2019, the FDA approved atezolizumab (anti-PD-L1) for the first time in the treatment of TNBC. The limitation of immunotherapy as a personalized therapy is that only a few patients benefit from it (Gil Del Alcazar et al., 2020). Therefore, it is necessary to provide a biomarker other than molecular subtypes to better realize the potential of immunotherapy in the treatment of breast cancer. In our article, we further revealed two subgroups in breast cancer patients based on 24 m6A regulators, and they present distinct immune infiltration characteristics. Recent bioinformatics studies have attempted to use m6A to classify breast cancer subtypes with different immune landscapes (He et al., 2021). However, no studies have screened m6A-related mRNA panels to predict the risk of tumor recurrence and survival outcomes. In our study, we confirmed that low-m6aRiskscore group manifest an immune activation phenotype, while high-m6aRiskscore group showed an immunosuppressive phenotype characterized by high M2 macrophages, low CD8^+^ T cells, High Treg cells and low CSF1, and high CSFR1. The poorer prognosis and lower immunogenicity further proved the characteristics of poor immune infiltrations. Interestingly, the PD-1 related genes in the high-m6aRiskscore group are lower than those in the low-m6aRiskscore group. A bioinformatics study also reached the same seemingly contradictory results. They found that the content of Treg in the immune-activated subgroup was strangely higher, but the article did not give much discussion (He et al., 2021). We presume that this result suggests that the immune escape of tumors under this classification system is mainly caused by the down-regulation of neoantigen peptide loading genes including MHC class I, rather than the inhibitory effect of PD-L1 immune checkpoints.

According to previous studies, there are multiple mechanisms in the progression of breast cancer to gradually suppress the immune environment. The first is the role of immune checkpoints such as PD-L1 and CTLA-4. PD-L1 and CTLA-4 can inhibit T cell activation until T cell exhaustion occurs. (Zhao et al., 2019), CTLA-4 also mediates the inhibitory effect of Tregs cells. (Zhang et al., 2021). Secondly, HER2 itself can trigger an anti-tumor immune response in tumors amplified by ERBB2. In some tumors, Th1 cells have impaired anti-HER2 ability and thus lead to immune escape (Datta et al., 2015). Finally, it is the down-regulation of HLA-like neoantigen peptide genes that are common in cancer cells. Most HLA class I antigen-processing machinery defects ( >75%) are caused by epigenetic mechanisms or signal transduction disorders, so we can design reasonable strategies to correct them knowing that this change is non-structural (Maggs et al., 2021). At present, for breast cancer with different characteristics, different types of immunotherapies have emerged, such as immune checkpoint inhibitors, mRNA vaccines, chimeric antigen receptor-modified T cells (CAR-T), and related nano-positioning technologies. Many ongoing breast cancer clinical trials are testing cancer vaccines, and the HER2-based targeted DC vaccine has achieved some results (Adams et al., 2019; Fennemann et al., 2019).

The comprehensive analysis also showed that m6aRiskscore is an independent prognostic biomarker for breast cancer. In terms of molecular typing, HER2-enriched and TNBC are the targets of immunotherapy clinically, which all present a higher m6aRiskscore. In addition, through the validation of internal and external immunotherapy datasets, we found that the m6ARiskscore of patients who responded to immunotherapy was elevated, which verified its predictive value. Taken together, these findings suggest that m6aRiskscore is significantly correlated with dominant immune cell type, immunogenicity, and molecular typing, helping to provide guidance for immunotherapy.

It is undeniable that the limitation of our studies must be mentioned. Further *in vitro* and *in vivo* experiments are needed to probe into the mechanism of m6A modification. In order to explore the applicability of our model, more independent BC cohorts are demanded to verify the prognostic value of the m6aRiskscore model.

## Conclusion

In conclusion, m6aRiskscore comprehensively evaluates the individual methylation modification patterns of breast cancer patients and their corresponding TME characteristics thus providing us a more effective guide for clinical practice. We also proved that m6aRiskscore could be used to assess the clinicopathological characteristics of patients, including tumor inflammation, clinical stage, molecular subtype, and response to immunotherapy. Similarly, we could use m6aRiskscore as an independent prognostic biomarker for predicting patient survival. Our findings provide new ideas for identifying different tumor immunophenotypes and facilitating personalized cancer immunotherapy in the future.

## Data Availability

The original contributions presented in the study are included in the article/Supplementary Material, further inquiries can be directed to the corresponding authors.

## Supplementary Material

Supplementary Figure S1 Cluster1 exhibited active protein transcription and post-translational modification processes, while Cluster2 was enriched in many apoptosis-related pathways.(A-C) Gene set enriched in Cluster1. (D-E) Gene set enriched in Cluster2.

Supplementary Figure S2 Some of the immune activation and immune-checkpoint genes were differentially expressed in cluster1/2.(A-E) The expression level of immune-related genes in cluster1/2.

Supplementary Figure S3 Low-m6aRiskscore group was enriched in immune response-related pathways, while the high-m6aRiskscore group was enriched with many malignant hallmarks of cancer.(A-B) Gene set enriched in low-m6aRiskscore. (C-E) Gene set enriched in the high-m6aRiskscore group.

## Abbreviations

AUC, Area under the curve; DEGs, Differentially expressed genes; GEO, Gene-Expression Omnibus; GSEA, Gene set enrichment analysis; GDC, Genomic Data Commons; GS, Gene significance; HR, Hazard Ratios; HLA, Human leukocyte antigen; ICB, Immune checkpoint blockade; m6A, N6-methyladenosine; MM, Module membership; MHC, Major histocompatibility complex; OS, overall survival; PCA, Principal component analysis; ROC, Receiver operating characteristic; TCGA, The Cancer Genome Atlas; TME, Tumor microenvironment; TCGA-BRCA, Cancer Genome Atlas-Breast Cancer; TOM, Topological overlap matrix; Tregs, T cells regulatory; Th2, T helper 2; WGCNA, Weighted correlation network analysis.
